# Supplementary figures and images for: Genome-Wide Analysis Reveals Transcription Factors Regulated by Spider-Mite Feeding in Cucumber (Cucumis sativus)
Source: Plants (Basel). 2020 Aug 11;9(8):1014. doi: 10.3390/plants9081014 (PMC7465836; doi:10.3390/plants9081014)

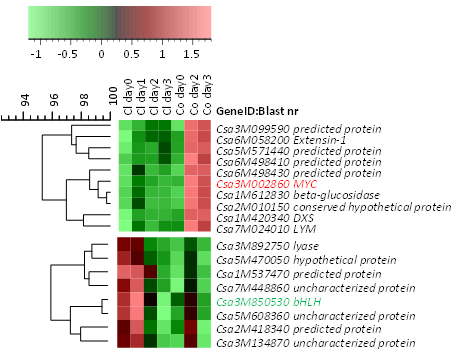

Supplement: Supplementary file 1 [file plants-09-01014-s001.zip › plants-879289-supplementary.tif]
